# Immunotherapy for Merkel cell carcinoma: a turning point in patient care

**DOI:** 10.1186/s40425-018-0335-9

**Published:** 2018-03-23

**Authors:** Isaac S. Chan, Shailender Bhatia, Howard L. Kaufman, Evan J. Lipson

**Affiliations:** 1 Department of Oncology, Johns Hopkins University School of Medicine, Sidney Kimmel Comprehensive Cancer Center, and Bloomberg-Kimmel Institute for Cancer Immunotherapy, Baltimore, MD USA; 2 Department of Medicine/Medical Oncology, University of Washington and Fred Hutchinson Cancer Research Center, Seattle, Washington, USA; 3 Department of Surgery, Massachusetts General Hospital, Boston, MA USA; 4 Melanoma and Cancer Immunology Programs, Johns Hopkins University School of Medicine, 1550 Orleans Street, Room 507, Baltimore, MD 21287 USA

## Abstract

Merkel Cell carcinoma (MCC) is a rare but aggressive cancer, with an estimated disease-associated mortality as high as 46%. MCC has proven to be an immunologically responsive disease and the advent of immune checkpoint inhibitors has changed the treatment landscape for patients with advanced MCC. In this review, we discuss the rationale for the use of immune checkpoint inhibition, review current single agent therapies tested in and approved for MCC, and discuss emerging immunotherapeutic options for these patients.

## Background

Merkel cell carcinoma (MCC) is a neuroendocrine-like tumor of the skin. First described in 1972, it is a rare diagnosis, with an annual incidence of approximately 0.6 out of 100,000 persons in the United States [1]. A high index of suspicion is required for diagnosis given its rarity and often inconspicuous presentation as a small, asymptomatic ulcerated, cystic or acneiform lesion [2]. The incidence of the disease has quadrupled since 1986, perhaps due the development of more sophisticated diagnostic tools, an aging population, and increasing use of therapeutic immunosuppression [3, 4]. Age, immunosuppression, and sun exposure remain the largest risk factors for this deadly disease, with an estimated disease-associated mortality of 33% to 46% [5]. In patients with localized MCC, the five-year overall survival rate is 55.6%. In patients with advanced disease, historical five-year survival are 35.4% for those with regional nodal disease and 13.5% for those with distant metastases [6]. These survival rates reveal the historical lack of effective treatment options for patients with MCC [7]. However, recent advances in our understanding of the biology of MCC have created opportunities for novel therapeutic strategies and hope for improving treatment efficacy. For example, the discovery of the oncogenic Merkel cell polyomavirus (MCPyV) that is associated with approximately 80% of MCC cases has led to further investigations into whether dysregulated immune surveillance plays a role in MCC pathogenesis, and how best to generate anti-tumor immunity [8]. Recent results from clinical trials of immune checkpoint inhibitors suggest that these therapies could improve treatment outcomes by unleashing anti-tumor immunity against an immunogenic tumor. In this review, we discuss the spectrum of therapeutic options for MCC and the pivotal role that immune checkpoint inhibition might play in improving patient outcomes.

Patients with primary, or localized MCC, which accounts for 65–70% of patients at diagnosis, typically undergo surgical resection followed by adjuvant radiotherapy to prevent recurrence at the primary site and involved regional lymphatics [6]. Even after definitive therapy of stage I and II disease, the potential for recurrence is high, with recurrence rates of 35% at three years [9]. In a case series of 237 MCC patients with local or regional disease, median time-to-recurrence was 9 months (range, 2–70 months) and 91% of the recurrences occurred within 2 years of initial diagnosis [10]. Study data do not support the routine use of adjuvant systemic chemotherapy for high-risk resected MCC and, therefore, adjuvant chemotherapy is not included in the NCCN guidelines. Without evidence demonstrating a clear survival benefit, the risks of immunosuppression, toxicity and diminished quality of life are not justified [11].

Until recently, chemotherapy has been a mainstay of therapy for patients with advanced MCC. Because MCC bears similarity to small cell lung cancer (SCLC), another neuroendocrine tumor, chemotherapy regimens used to treat MCC were modeled after regimens used in SCLC [12]. Early cases were treated with drugs such as cyclophosphamide, doxorubicin and vincristine, but reports described limited efficacy [13]. More recently, platinum agents in combination with etoposide became the preferred chemotherapy regimen. Although MCC is often chemosensitive initially, responses are generally not durable. For example, one retrospective study reported an overall response rate of 55%, but a median progression free survival of only 94 days [14]. Thus, there is a great need for discovering and testing new therapeutic options.

### The emergence of immune checkpoint inhibition

Immune checkpoints are a cadre of molecules regulating T cell activation and proliferation which may become dysregulated or co-opted and allow the tumor to escape immune surveillance [15]. Discoveries in the 1980s and 1990s brought a greater understanding of the molecular underpinnings of self-tolerance and the ways in which immune checkpoint molecules control immunoregulatory signaling and T cell responses [16]. These discoveries led to the development of clinical agents targeting immune checkpoint ligands and receptors. The first of such inhibitors targeted CD152 (cytotoxic T-lymphocyte-associated antigen 4; CTLA-4). One such drug, ipilimumab, was the first in its class to demonstrate an improvement in overall survival in a clinical trial for patients with metastatic melanoma, which led to its approval by the FDA in 2011 [17]. Since then, other drugs have entered the market that target programmed cell death protein 1 (PD-1), another immune checkpoint receptor, or one of its associated ligands (PD-L1), and are now FDA-approved for a variety of cancers [18]. The success of immune checkpoint inhibitors in several cancer types and the immune susceptibility of MCC has renewed hope for developing more effective treatment options for patients with MCC.

### The immune system and Merkel cell carcinoma

It has been long suspected that immune dysregulation plays a role in the development of MCC. Clinically, it was observed that chronically immunosuppressed patients, such as organ transplant recipients or those with HIV or B-cell malignancies, were at increased risk for developing MCC [19–22]. Early histological reports of primary MCC tumors demonstrated lymphocytic infiltration, evidence of MCC’s immunogenic biology [23, 24]. More recently, tumor-infiltrating lymphocytes were found to correlate with a better prognosis, a finding which has been confirmed by genomic analysis of primary MCC tumors [25, 26]. Of note, patients with an unknown primary lesion (e.g., those who present with a nodal metastasis only) have a better prognosis than those with a known primary lesion, suggesting that an immune-based response at the primary site leads to improved immunological tumor control overall [27–29].

In 2008, Feng and colleagues described an oncogenic Merkel cell polyomavirus (MCPyV), present in about 80% of MCC tumors. MCPyV creates a large T antigen that inactivates tumor suppressors p53 and RB. This discovery not only identified a causative factor for MCC, but also suggested a role for immune evasion in MCC’s oncogenesis [30, 31]. Viral antigens are expressed in MCC tumor cells and there is strong evidence for their recognition by innate and adaptive (i.e., cellular and humoral) immune elements [32]. Virus-negative MCCs may also be immunogenic, perhaps based on their high tumor mutation burden and neoantigens created as a result of ultraviolet light exposure [33]. However, despite their inherent immunogenicity, MCC tumors are able to evade the immune system through multiple mechanisms including the expression of immune checkpoint molecules. Notably, over 50% of Merkel-cell carcinomas express PD-1 on tumor-infiltrating lymphocytes and express PD-L1 on tumor cells [34]. The totality of these data provided a strong rationale for testing immune checkpoint blockers in patients with advanced MCC.

### Immune checkpoint inhibition in MCC

Pembrolizumab was the first immune checkpoint inhibitor to demonstrate objective tumor regressions in patients with MCC [35]. Pembrolizumab is a humanized monoclonal antibody against PD-1 and is now FDA-approved for use in patients with a variety of cancers. In a phase 2, single-arm, multicenter study (ClinicalTrials.gov number NCT02267603), patients with advanced MCC who had not previously received systemic therapy were treated with pembrolizumab 2 mg/kg every three weeks for a maximum of two years or until disease progression, dose-limiting toxicity, or complete response. Out of 26 patients, 4 experienced a complete response (CR) and 10 had a partial response (PR), for an ORR of 56%. At 6 months, the progression-free survival rate was 67% and the duration of response ranged from 2.2 months to at least 9.7 months. 86% of responses were ongoing at last follow-up. (Table 1) These results prompted the addition of pembrolizumab for the treatment of disseminated MCC to the National Comprehensive Cancer Network (NCCN) guidelines [36]. Interestingly, objective regression was observed in both virus-positive and virus-negative tumors. PD-L1 expression did not seem to correlate with a higher likelihood of response to treatment as it has in other tumors [37].

**Table 1:** Activity of PD-1-pathway-targeted agents in patients with advanced Merkel cell carcinoma

Drug name	Drug class	N	Number of prior systemic therapies	ORR (%)	Reference
Avelumab	Anti-PD-L1	88	1–4	33	[40]
		29	0	65	[53]
Nivolumab	Anti-PD-1	15	0	73*	[44]
		10	1–2	50*	
Pembrolizumab	Anti-PD-1	26	0	56	[35]

*RECIST v1.1, investigator assessed

In March 2017, a PD-L1 monoclonal antibody, avelumab, became the first FDA-approved treatment for MCC [38]. Approval was based on data from an open-label, single-arm, multi-center phase 2 clinical trial (JAVELIN Merkel 200; NCT02155647) [39]. In this study, 88 patients with advanced MCC who had progressed after receiving chemotherapy received avelumab 10 mg/kg every 2 weeks. Updated outcomes at a median follow-up duration of 16.4 months revealed an ORR of 33%, including 10 CRs and 19 PRs [40]. (Table 1) Similar to the pembrolizumab trial, objective responses were observed regardless of PD-L1 expression or MCPyV status. Responses were ongoing in 21/29 patients (72.4%) at last report. A separate, parallel cohort has been actively recruiting chemotherapy-naïve patients with advanced MCC. Preliminary data among 25 patients with > 6 weeks of follow-up demonstrated an unconfirmed ORR of 64% [41]. When compared to historical trials of patients with advanced MCC receiving chemotherapy, the durability of responses to avelumab appears substantially superior [14, 42, 43]. (Table 2).

**Table 2:** Progression-free survival (PFS) outcomes for previously-treated patients with advanced MCC after treatment with avelumab (anti-PD-L1) compared with PFS rates from previous chemotherapy trials (historical controls)

Anti-neoplastic agent(s)	Median PFS, months (95% CI)	PFS rate at 12 months, % (95% CI)	Reference
Avelumab (*N* = 88)	2.7 (1.4–6.9)	30 (21–41)	[39]
Cowey 2017 (*n* = 20)	2.1 (1.0–3.2)	0	[42]
Becker 2016 (*n* = 34)	3.0 (2.6–3.1)	0	[43]
Iyer 2016 (*n* = 30)	2.0 (NA [range: 0.4–11.6])	0*	[14]

The most common second-line chemotherapy was topotecan. NA, not available; *based on PFS range (11–354 days) and Kaplan-Meier PFS estimates

Nivolumab is another monoclonal PD-1 antibody with clinical activity in advanced MCC. As part of the phase 1/2 multiple cohort CheckMate 358 study (NCT02488759), 25 patients with both treatment-naïve and previously-treated, MCPyV-positive or -negative, advanced MCC were enrolled and treated with nivolumab 240 mg every 2 weeks [44]. Among 25 patients, with a median follow-up of 51 weeks (range: 5–63 weeks), investigators observed a 64% ORR. Arithmetically, the ORR was higher among the 15 treatment-naïve patients (73%) compared with the 10 previously-treated patients (50%), though these numbers are too small to reach statistical significance. The median duration of response was not reached. Consistent with findings in the two trials described above, objective responses occurred independent of PD-L1 expression and MCPyV status. Expansion cohorts on this trial are investigating the activity and safety of nivolumab in combination with ipilimumab or relatlimab (BMS-986016, anti-LAG-3) in patients with advanced MCC or other virus-associated cancers. Of note, ipilimumab monotherapy has demonstrated durable anti-tumor activity in small case series of 5 chemotherapy-naïve patients with metastatic MCC [45].

### Adverse reactions

The safety profiles of the PD-1/PD-L1 antibodies administered to patients with MCC appear similar to those from previous trials involving patients with other tumor types. Immune-mediated adverse reactions observed on the trials described above included adrenal insufficiency, colitis, hepatitis, myocarditis, nephritis, pneumonitis, thyroiditis, and transaminitis, among others. Of note, infusion-related reactions were observed with administration of avelumab, so premedication with an antihistamine and acetaminophen prior to the first four infusions of avelumab is now recommended [46]. In the avelumab trial, there were 5 grade 3 treatment-related adverse events reported in 4 (5%) patients, including two cases of lymphopenia, and one case each of isolated elevations in serum creatine phosphokinase, alanine and aspartate aminotransferase (AST/ALT), or cholesterol. There were no treatment-related grade 4 adverse events or deaths observed in the trial [37]. Of patients receiving pembrolizumab, grade 3 or 4 treatment-related adverse events were observed in 15% of patients [35]. Grade 4 events included myocarditis and elevated AST/ALT. Similarly, in CheckMate 358, grade 3 or 4 treatment-related adverse events were reported in 20% of patients and 12% had adverse events that led to nivolumab discontinuation [44].

## Conclusions and future directions

Immunomodulatory therapies have had a profound impact on the cancer treatment landscape, and MCC is no exception. Indeed, response rates to single-agent immune checkpoint inhibition seem to compare favorably to those of other tumor types [47]. With the recent FDA approval of avelumab for previously-treated advanced MCC, patients with MCC now have a new treatment option beyond chemotherapy. The results of the trials described above led to the inclusion of avelumab, pembrolizumab and nivolumab in the January 2018 NCCN guidelines as preferred treatment options for patients with disseminated disease [36]. Although data are still preliminary, it appears that rates of MCC regression in treatment-naïve patients treated with PD-1/PD-L1-pathway blockers may exceed those of patients who were previously treated. These findings require validation in larger patient cohorts, but suggest that immune checkpoint blockade may be most efficacious when used in the first-line setting. Furthermore, responses appear to be durable, unlike those seen with cytotoxic chemotherapy and hence, these agents are becoming the new standard-of-care for treating patients with metastatic or unresectable MCC.

The immunogenic characteristics demonstrated by both MCPyV-positive and -negative Merkel cell tumors perhaps underlie its sensitivity to immuno-oncology agents. Ongoing and future trials aim to capitalize on this phenotype by interrogating and manipulating the tumor microenvironment and host immune system in order to develop more effective combinatorial regimens. One such trial combines localized radiotherapy or recombinant interferon beta and avelumab with or without cellular adoptive immunotherapy for patients with metastatic MCC. Both radiation and interferon beta can enhance the host immune response by upregulating MHC class I molecules. Combining either approach with polyclonal CD8+ T cells and a PD-L1 blocker (avelumab) may enrich the immune microenvironment by expanding molecular immune targets, allowing for anti-tumor T cell activation (NCT02584829). Another phase 2 study (NCT02465957) seeks to combine activated NK-92 natural killer cell infusions with ALT-803 (interleukin-15) in patients with advanced MCC. One emerging area of investigation is focused on determining when administration of immune checkpoint inhibition is most effective. For example, several clinical trials are investigating the utility of immune checkpoint blockers in the adjuvant setting (e.g., NCT02196961, NCT03271372) administered with or without radiotherapy. Combining these modalities may provide synergistic anti-tumor activity for patients with stage III MCC (i.e., regional nodal metastases), for whom adjuvant RT alone has, historically, not led to improvements in overall survival [48]. This approach follows successful adjuvant trials in other locoregionally advanced cutaneous malignancies (e.g., stage III melanoma) [49]. The neoadjuvant setting also provides an opportunity for administration of immune checkpoint inhibition. Early data from clinical trials suggest benefit, including in patients with melanoma [50]. Other therapies on the horizon include trials combining intralesional T-VEC (talimogene laherparepvec), an oncolytic, recombinant herpes simplex type-1 virus-based agent, with radiotherapy (NCT02819843) or nivolumab (NCT02978625). The FDA’s recent approval of tisagenlecleucel [51], a chimeric antigen receptor T cell (CAR-T) therapy, for patients with B-cell precursor acute lymphoblastic leukemia underscores the potential for this immune-based anti-cancer strategy. Given the role of MCPyV in driving MCC carcinogenesis, a future treatment approach may involve administration of genetically-modified CAR-T cells against MCPyV antigens.

Because MCC is a rare cancer, our understanding of the disease biology and the utility of novel therapies seems best strengthened by conducting international, multi-center, and cooperative group trials using novel study designs [52]. In the last few years, advances in our understanding of how immunotherapies can treat patients with MCC have brought hope and optimism to cancer researchers, clinicians and patients alike, and have laid a foundation for the continued development of safe and effective treatment regimens for patients with this rare, deadly disease.

## Abbreviations

ALT: alanine aminotransferase
AST: aspartate aminotransferase
CAR-T: chimeric antigen receptor T-cell
CTLA4: Cytotoxic T-lymphocyte-associated protein 4
DoR: Duration of response
MCC: Merkel cell carcinoma
MCPyV: Merkel cell polyomavirus
NCCN: National Comprehensive Cancer Network
ORR: Objective response rate
PD1: Programmed cell death protein 1
PDL1: Programmed cell death-ligand 1
PFS: Progression-free survival
